# A Simplified *In vitro* Experimental Model Encompasses the Essential Features of Sleep

**DOI:** 10.3389/fnins.2016.00315

**Published:** 2016-07-07

**Authors:** Ilaria Colombi, Federico Tinarelli, Valentina Pasquale, Valter Tucci, Michela Chiappalone

**Affiliations:** ^1^Department of Neuroscience and Brain Technologies, Istituto Italiano di TecnologiaGenova, Italy; ^2^Dipartimento di Neuroscienze, riabilitazione, oftalmologia, genetica e scienze materno-infantili (DINOGMI), Università degli Studi di GenovaGenova, Italy

**Keywords:** cortical culture, microelectrode arrays, homeostasis, spike train analysis, local field potentials, gene expression

## Abstract

In this paper, we show that neuronal assemblies plated on Micro Electrode Arrays present synchronized, low frequency firing patterns similar to *in vivo* slow wave oscillations, which are a key parameter of sleep-like state. Although neuronal cultures lack the characteristic high-frequency waves of wakefulness, it is possible to modulate their spontaneous firing pattern through the administration of specific neurotransmitters such as acetylcholine. We thus stimulated the cortical cultures with an agonist of acetylcholine receptor, Carbachol, which caused a desynchronization of the spontaneous firing of the cultures. We recorded and monitored the cultures for a period of over 31 h. We analyzed the electrophysiological signals by exploiting novel methodological approaches, taking into account the different temporal scales of the recorded signals, and considering both spikes and local field potentials. Supporting the electrophysiological analysis results, gene expressions of targeted genes showed the activation of specific markers involved in sleep-wake rhythms. Our results demonstrate that the Carbachol treatment induces desynchronization of neuronal activity, altering sleep-like properties in an *in vitro* model.

## Introduction

Classically, sleep is defined by electrophysiological signatures measured primarily in *in vivo* systems and it is characterized by the interaction of two different biological processes, circadian and homeostatic (Borbély, 1982). The circadian process is self-sustained, mainly related to cell-autonomous mechanisms. Its clock governs the daily cycle of many biological activities, such as the control of body temperature and hormone secretion (Stokkan et al., 2001; Reppert and Weaver, 2002). The homeostatic process depends on the prior wakefulness periods and tracks sleep need. It can be modeled by a mathematical function that increases during wakefulness and decreases during sleep, thereby describing the pressure for sleep across a circadian cycle (Borbély, 1982; Daan et al., 1984; Franken et al., 2001). Many studies have demonstrated a strong interplay between circadian and homeostatic components of sleep (Franken et al., 2001). Interestingly, among the core clock genes, *PER2* has shown a dual role in both the cellular clock machinery and the homeostatic control of sleep. Many mouse studies have demonstrated that *Per* genes control slow wave activity following sleep loss (Kopp et al., 2002; Shiromani et al., 2004). Moreover, new molecular targets and epigenetic regulatory mechanisms are emerging as novel players in the control of sleep and its homeostatic control (Tinarelli et al., 2014). Besides circadian control, sleep-like activity continues to be identified as an emerging property of neuronal networks.

Sleep can be characterized by the alternation of two different physiological and behavioral states, each of them with distinct electrophysiological features: the rapid eye movement (REM) sleep and the non-rapid eye movement (NREM) sleep. Specific characteristics of REM/NREM sleep phases depend on the complexity of the Central Nervous System. However, there are common shared traits among species, such as specific properties associated with the frequency spectrum of distinct sleep states (Lo et al., 2004; Phillips et al., 2010; Brown et al., 2012). In particular, the electroencephalographic (EEG) activity is characterized by two different patterns: slow oscillations in the delta frequency range (1–4 Hz) during the deep sleep (NREM), and rapid, low voltage waves, such as beta (11–30 Hz) and gamma (30–120 Hz), during wakefulness and REM sleep. There are electrophysiological similarities between awaking states and REM sleep (also defined “paradoxical sleep”), which in an *in vivo* system are compensated by additional features (e.g., muscle atonia and metabolic/thermoregulatory alterations) that allow their distinction. But the investigation of the neural network's dynamical proprieties during sleep states is difficult while studying *in vivo* experimental systems.

Over the last few years it has been proposed that electrophysiological markers for sleep cycles can be also derived from *in vitro* neuronal networks, similar to those present on *in vivo* recordings (Sengupta et al., 2011; Hinard et al., 2012). To this end, a lot of effort in current sleep science aims at understanding to which extent an *in vitro* system can recapitulate the essential features of a sleeping brain. Cortical cultures represent a simplified and easily accessible *in vitro* model of the rat cerebral cortex, used for studying principles of neurodynamics and neural coding (Marom and Shahaf, 2002; Chiappalone et al., 2007, 2008). Moreover, multisite extracellular recordings, by means of Micro-Electrode Array (MEA; Gross et al., 1977; Colombi et al., 2013), have the potential to reveal individual cell behaviors as well as network dynamics. Although neuronal assemblies plated on MEA show spontaneously synchronized, low frequency firing patterns (Van Pelt et al., 2004; Chiappalone et al., 2007; Corner, 2008), which resemble the slow wave oscillations that characterize NREM sleep *in vivo* (Steriade et al., 1993; Rector et al., 2005), the lack of the awake state counterpart limited the investigation of the main physiological aspects of sleep. To overcome this problem, that is, to mimic wake-like states in neuronal networks and to possibly reproduce the homeostatic balance between sleep and wake states in dissociated neuronal networks, a recent combination of pharmacological treatment was proposed (Hinard et al., 2012). The latter study has provided pivotal evidence that dissociated neurons *in vitro* could reproduce sleep-like properties. It becomes now important to dissect all possible components that play a role in the sleep-like states of dissociated neurons.

In our study we attempted to study sleep-like properties *in vitro* by manipulating the electrophysiological activity of dissociated neurons by simply altering the membrane excitability (le Feber et al., 2014). In particular, we took advantage of the functional role of neurotransmitter acetylcholine. *In vivo* acetylcholine promotes high-frequency oscillatory activity usually present in wakefulness and REM sleep (Deurveilher and Semba, 2011; Platt and Riedel, 2011; Brown et al., 2012). Therefore, we stimulated our *in vitro* neuronal networks using the cholinergic agonist Carbachol (CCh), inspired by already published works (Tateno et al., 2005; Corner, 2013). Remarkably, we observed for the first time that CCh treatment affects both the high and the low frequency components of the signal, causing an abolishment of the synchronous activity and then potentially altering sleep-like electrophysiological properties.

## Materials and methods

### Cell culture

Dissociated neuronal cultures were obtained from cortices of embryonic rats at gestational day 18 (pregnant Sprague-Dawley female rats delivered by Charles River Laboratories, Lecco, Italy). All experimental procedures and animal care were conducted in conformity with institutional guidelines, in accordance with the European legislation (European Communities Directive of 24 November 1986, 86/609/EEC) and with the NIH Guide for the Care and Use of Laboratory Animals. The procedures for preparing neuronal cultures are described in detail in previous studies (Frega et al., 2012; Colombi et al., 2013). Briefly, the cerebral cortices of 4–5 embryos were dissected out from the brain and dissociated by enzymatic digestion in 5 ml of trypsin 0.125% and HBSS, diluted DNAsi 0.25 mg/ml (Sigma-Aldrich, Saint Louis, Missouri, USA) at 37°C for 30 min. Trypsin digestion was blocked using 5 ml of Neurobasal Medium (Thermo Fisher Scientific, Waltham, MA, USA) containing 2% of B27 supplement, 1% penicillin/streptomycin, 1% L-glutamine (Thermo Fisher Scientific, Waltham, MA, USA), plus 10% of heat inactivated FBS (Thermo Fisher Scientific, Waltham, MA, USA). Cells were centrifuged 5 min at 1200 rpm and then resuspended by pipetting in 2–3 ml of complete Neurobasal medium plus FBS. Cells debris was removed centrifuging at 700 rpm for 7 min. Neurons were then resuspended in complete culture medium without FBS and counted with trypan blue dye (Sigma-Aldrich). Cells were plated onto 60-channel MEAs previously coated with poly-D-lysine and laminin to promote cell adhesion (final density around 1200 cells/mm^2^) and maintained with 1 ml of nutrient medium (i.e., serum-free Neurobasal medium supplemented with B27 and Glutamax-I). They cells were finally placed in a humidified incubator having an atmosphere of 5% CO2–95% air at 37°C. Half of the medium was replaced weekly.

### Micro-electrode array recordings

**Figure 1:**
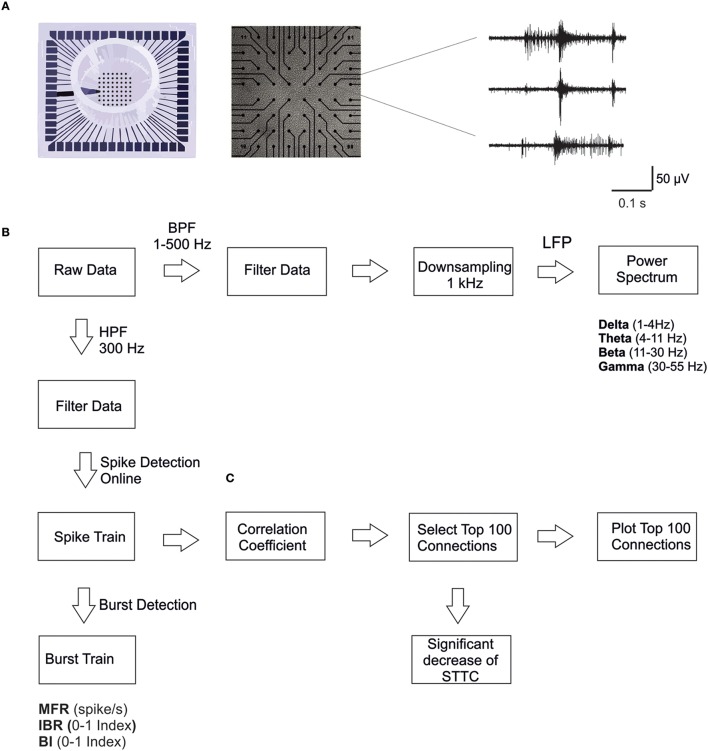
**MEA electrodes layout and data analysis. (A)** A 60-channel-MEA (Multichannel Systems MCS, Reutlingen, Germany) with the standard electrode layout (8 × 8) plated with cortical cultures. On the right, typical traces recorded from three microelectrodes upon high pass filtering at 300 Hz, to selectively consider MUA only. **(B)** Scheme of the data analysis workflow. We performed spike and burst detection on the high pass filtered signal using MC_Rack software (MCS). Before computing power spectral density, we down-sampled the signal to 1 KHz, previously filtered using a low pass filter (500 Hz). **(C)** Scheme of the correlation analysis: we considered only the strongest 100 STTC (Spike Time tiling coefficient) during the basal recording (i.e., 7 h), in order to select only the most significant correlations. Then we calculated the percentage of decrease in the STTC values. Therefore, we plot connectivity maps of the 100 STTCs.

Planar microelectrodes are arranged in an 8 × 8 layout, excluding corners and one reference electrode, for a total of 59 TiN/SiN planar round recording electrodes (30 μm diameter; 200 μm center-to-center inter electrode distance; Multichannel Systems, MCS, Reutlingen, Germany; Figure 1A). One recording electrode was replaced by a bigger ground electrode. The activity of all cultures was recorded by means of the MEA60 System (MCS). The signal from each channel was sampled at 10 kHz and amplified using a Multichannel System amplifier with a bandwidth of 1 Hz–3 kHz. Each recorded channel was acquired through the data acquisition card and on-line monitored through MC_Rack software (MCS). To reduce thermal stress of the cells during the experiment, MEAs were kept at 37°C by means of a controlled thermostat (MCS) and covered by PDMS caps to avoid evaporation and prevent changes in osmolarity.

### Experimental protocol

**Figure 2:**
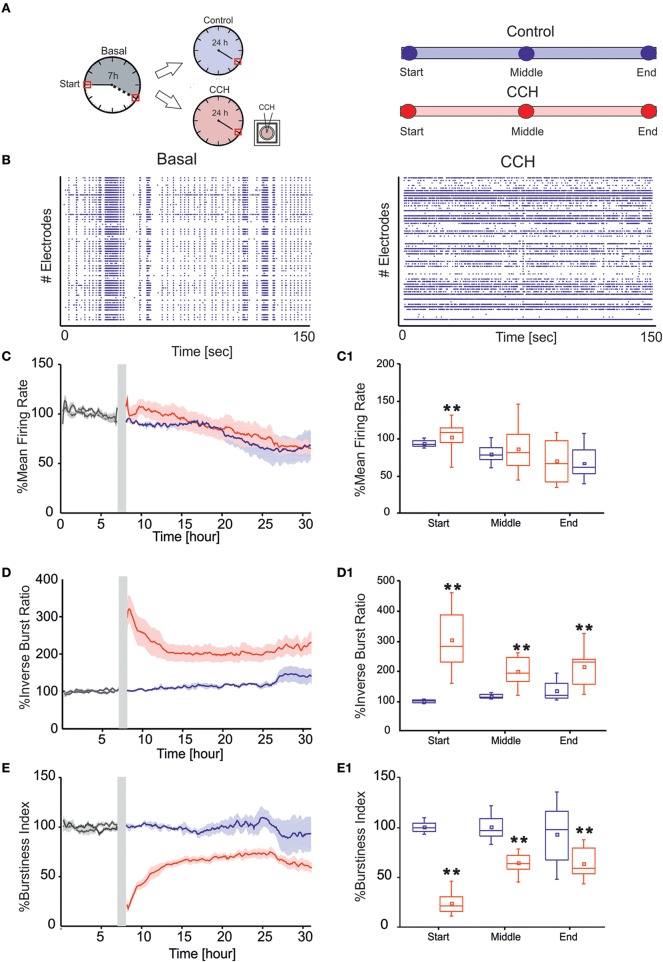
**MUA analysis. (A)** Left panel: Schematic representation of experimental protocol using chronic treatment of Carbachol (CCh). Cortical cultures are recorded for 7 h in basal condition, followed by 24 h of recording, upon addition of 20 μM CCh. Right Panel: Time point of 1 h considered for the evaluation of the networks parameters for each of the two different experimental condition. **(B)** Raster plots showing 150 s of spontaneous (left) and after CCh application (right) for a representative experiment (each small dot represents a spike, each row an electrode). In the basal condition, the cortical culture shows a synchronized activity completely disrupted after the CCh treatment. **(C)** MFR of cultures (left) recorded without (blue line: *N* = 4; cultures age 42 ± 5 DIV) and with stimulation (red line: *N* = 7; cultures age 34 ± 2 DIV). **(C1)** Box plot of MFR (right) during control experiments (blue box) and during CCh treatment (red box at three different 1-h time intervals: start, middle, and end for each experimental condition. In the analyzed group of experiments, the MFR of cultures upon drug administration changed significantly only in the first 1 h, increasing by 9.55% respect to the control experiments in the same interval of time **(D)** IBR of cultures (left) recorded without (blue line) and with stimulation (red line). **(D1)** Box plot of IBR (right) during control experiments (blue box) and during CCh treatment (red box). In the analyzed group of experiments, the IBR of cultures upon drug administration significantly increased compared to the control experiments during the entire recording. In particular, during the first 1 h, IBR increased by 200% compare to the control in the same period of time. **(E)** BI of cultures (left) recorded without (blue line) and with stimulation (red line). **(E1)** Box plot of BI (right) during control experiments (blue box) and during CCh treatment (red box). In the analyzed group of experiments, the BI of cultures significantly decreased upon drug administration compared to the control experiments during the entire recording. BI in the first 1 h decreased by 76% respect to the control in the same interval of time. Bold lines and shaded regions **(C–E)** correspond to mean ± SEM. Gray line corresponds to the basal recording for the two experimental conditions. Gray-shaded area indicates 10 min of recording stop, after CCh addition, to prevent experimental bias due to drug release in the medium. For each box plot **(C1–E1)**, the small square indicates the mean, the central line illustrates the median and the box limits indicate the 25th and 75th percentiles. Whiskers represent the 5th and the 95th percentiles. Statistical Analysis was carried out using Mann–Whitney comparison test, ^**^p < 0.01.

The general protocol included 7 h of recording in culture solution (Neurobasal +2% B27+1% Glutamax-I 200 μM + 1% Penicilin-Streptomycin sol.), defined as basal condition (Figure 2A). Carbachol (CCh) stock solution (100 mM) was prepared and diluted in DMSO-water, in order to obtain a final concentration of 20 μM (Tateno et al., 2005; Corner, 2008; Kaufman et al., 2012). The drug was added to the bath solution by directly pipetting in the medium (volume of 1 mL). Some of the cultures were exposed to CCh for 24 h, while for control experiment we recorded neuronal networks in culture solution for the same time temporal window. Since we noticed that mechanical perturbation due to the pipette injection in the medium could cause a temporary instability of the firing rate, we discarded the first 10 min at the beginning of the 24-h recording phase. The total number of experiments performed by following the above described protocol were: 4 for control (42 ± 5 Days *in vitro*, DIV) and 7 for CCh (34 ± 2 DIV). The two groups were not statistically different in terms of age of the tested cultures (*p*>0.05, Mann–Whitney's test).

### Data analysis

The typical extracellularly acquired signals from a local network of neurons are Local Field Potentials (LFPs) and Multi-Unit Activity (MUA). The LFPs, which constitute the low frequency components (< 300 Hz) of the raw signal, are believed to be generated by neuronal membrane currents in the local neighborhood of the recording electrode. MUA, which constitutes the high-frequency portion (>300 Hz) of the raw signal, represents the spiking of local neurons nearby the recording electrode (Burns et al., 2010; Buzsáki et al., 2012). For these reasons we analyzed the electrophysiological signals by exploiting novel methodological approaches, which can take into account both temporal scales of the recorded signals (i.e., LFPs and MUA). The different steps of the analysis are briefly reported in the following (Figure 1B).

#### MUA analysis

Firstly, extracellular signals were filtered online (MC_Rack software, MCS) by using a second-order Butterworth high-pass filter with cut-off frequency of 300 Hz, in order to select only the MUA component of the signal (Figure 1B). The online spike detection was performed (MC_Rack software, MCS) with hard threshold chosen as five times the standard deviation of the noise (−5σ_n_). To check for changes in the noise level during long recordings, we performed a *post-hoc* test for significant differences in the wide-band raw data standard deviation on a selected subset of control experiments. We found no significant changes in the standard deviation of the noise during the entire duration of the experiments. Afterwards spike trains were analyzed off-line using a custom software developed in Matlab (The Mathworks, Natick, MA, USA), named SpyCode (Bologna et al., 2010), which collects several tools for processing multi-channel neuronal recordings. We performed a custom burst detection as previously described (Chiappalone et al., 2005). Once spikes and bursts were detected, we were able to compute several parameters such as Mean Firing Rate, MFR (mean number of spikes over an interval of time [spike/s]), Inverse Burst Ratio, IBR (percentage of spikes outside bursts) and Burstiness Index, BI, index of the burstiness level of the network, as initially presented in (Wagenaar et al., 2005). The parameters extracted through burst and spike detection were normalized for each experiment with regard to the corresponding value of the reference (basal) activity for direct comparability. The same procedure has been described in previous papers from our group (Colombi et al., 2013).

#### Correlation analysis

First, we calculated the Spike Time Tiling Coefficient (STTC; Cutts and Eglen, 2014) between each pair of spike trains recorded from active channels (i.e., having MFR > 0.01 spike/s) every 1 h (Figure 1C). We only considered the largest 100 STTCs selected during the basal recording (i.e., 7 h), to identify only the most significant correlations. We then calculated the percentage of STTCs significantly decreasing during the control and the CCh application (i.e., decrease higher than 20%, mean + 3SD of the variations observed during basal recording). Finally, for the sake of clarity, we plotted the top 100 STTCs in the connectivity maps at different time points, both for the control and for the CCh experiments.

#### LFP analysis

Wide-band signals were first down-sampled to 1 kHz, after low pass filtering below 500 Hz to prevent aliasing (using a Hamming-window FIR filter of order 30; Leondopulos et al., 2012). Then we computed the power spectral density of the decimated signal (μV^2^/Hz), using Welch method [Windows = 5 s, overlap = 50%; DFT (Discrete Fourier Transform) points = 8192; df (frequency resolution) = 0.12 Hz; dt (temporal resolution) = 8.19 s; Figure 1B]. We only considered the lower frequency bands of the signal, which are of particular interest for studying the sleep-wake cycle, in particular delta (1–4 Hz), theta (4–11 Hz), beta (11–30 Hz), and gamma (30–55 Hz) bands. To characterize LFP, we calculated the power in each of those frequency bands.

We also plotted the different waves previously described (i.e., delta, theta, beta, and gamma), by filtering the LFP signal using FIR filters with different lengths (Leondopulos et al., 2012): 20,000 for delta (1–4 Hz), 10,000 for theta (4–11 Hz) and beta (11–30 Hz), and 5000 for gamma (30–55 Hz). All filtering operations were time compensated to account the delay introduced by the filter.

### Gene expression

Multi-well plates were coated using Poly-D-lysine 0.1 mg/ml (Sigma-Aldrich) and incubated overnight in a sterile incubator at 37°C and 5% CO2. Neurons were then plated at a concentration of 500,000 cells/well. Primary neurons were treated after DIV 30 (i.e., same age used for the MEA experiments) with 20 μM of Carbachol (Sigma-Aldrich) for 24 h. A wash out was then performed and the cells were put to recovery with conditioned medium for other 24 h prior to harvesting. Neurons were washed three times with ice-cold phosphate-buffered saline solution and lysed with 300 μl of Trizol (Life Technologies), followed by a phenol/chloroform extraction. Cell lysates were collected and 600 ng of RNA was retro-transcribed using ImpromII reverse transcription kit (Promega), according to manufacturer's specifications. Real Time-PCR was performed and analyzed as previously described (Tinarelli et al., 2014) in duplicate for each sample. All samples were normalized against b-actin. Primers used in the different Real Time-PCR experiments are reported in Table 1.

**Table 1:** **Primers used in the Real Time-PCR experiments**.

**Gene name**	**Primer sense**	**Primer reverse**
b-actin	TATGGAATCCTGTGGCATC	GTGTTGGCATAGAGGTCTT
Arc	TCTACACTCTCAGACCAT	ACACTTGATTTCTTGACTAC
Dlk-1	AAATAGATATTCGGGCTTGC	ATTCGTACTGGCCTTTCT
Fos	CATTACAGAGAGAAGAAACAAGT	TTCACGCACAGATAAGGT
Homer1-a	TAACCTGAAGACTCTCCTA	ACGAAGACAGACAGTATC
Per2	CATACCTTCACTGCTTCTT	AGTCTCCTCAAGTCCAAT

### Statistics

Data are expressed as mean ± standard error of the mean (SE). Statistical tests were performed to assess the significant difference among the experimental conditions. The normal distribution of data was assessed using Kolmogorov-Smirnov normality test. According to the distribution of data we performed either parametric (e.g., *t*-test) or non-parametric test (e.g., Mann–Whitney) test and *p* < 0.05 were considered significant. Statistical analysis was carried out by using OriginPro (OriginLab Corporation, Northampton, MA, USA).

## Results

In the present study we examined the long-term effect of CCh on the spontaneous network activity of mature cortical cultures grown over MEAs (Figure 1A). In particular, we compared the behavior of cortical networks with (*N* = 4 cultures) or without treatment (*N* = 7 cultures). In control experiments, the networks revealed a spontaneous, synchronized, and low-frequency (0.16–3 Hz) multi–unit burst activity, composed of network-wide brief (50–100 ms) bursts separated by periods of nearly complete quiescence or sparse, asynchronous action potentials (Maeda et al., 1995; Van Pelt et al., 2004; Wagenaar et al., 2005; Eytan and Marom, 2006; Chiappalone et al., 2007), as depicted in Figure 2B, left panel. Following CCh application, these activity patterns were strongly altered, in particular at the beginning of the administration. This resulted in a loss of regularity, and in fragmentation of burst structures, with many more isolated spikes, as shown in the raster plot of one representative experiment (Figure 2B, right panel) and in the measure of network synchronization described below (Figures 2D,E). In fact, with respect to the control, CCh increased significantly the percentage of out-burst spikes (Inverse Burst Ratio) during the entire recording (Figure 2D). This behavior is also confirmed by the BI (Burstiness Index) which significantly decreased with respect to the control experiments (Figure 2E) In contrast, the overall activity levels expressed by firing rate were significantly affected only in the first hour (Figure 2C). These effects are in agreement with previous reports (Tateno et al., 2005; Corner, 2008; Kaufman et al., 2012). The percentage of active channels remained high in both experimental conditions: 88.16% for the control experiment and 80.20% during CCh treatment. We calculated the percentage changes as follows:

$$\%Values=100\;*\;\frac{XEnd-Xstart}{Xstart}$$

**Figure 3:**
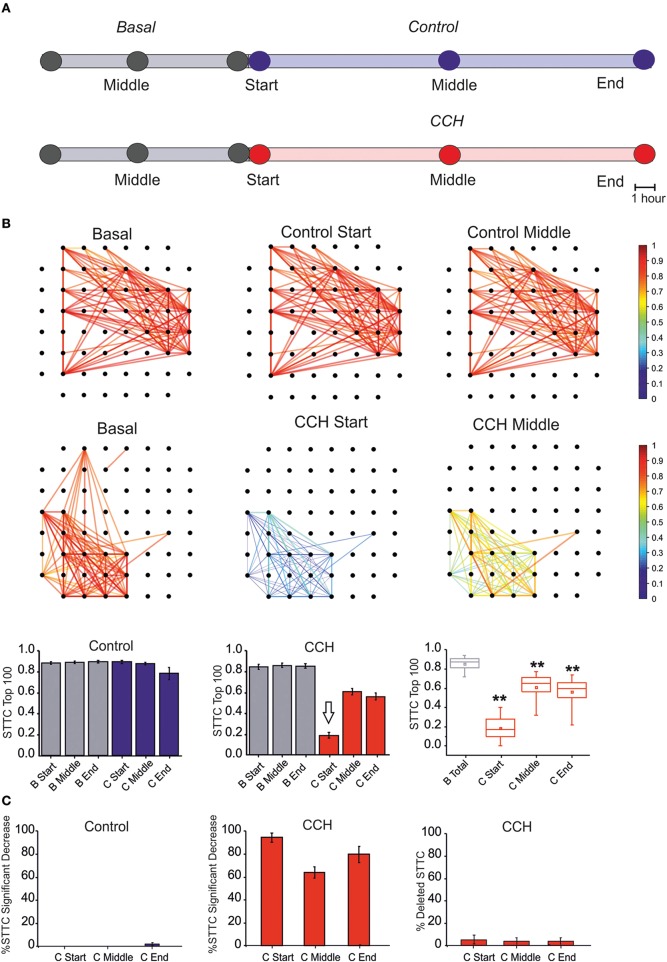
**Correlation Analysis. (A)** One-hour time intervals at three different times considered for the graph of the functional connectivity for each two different experimental conditions. **(B)** Plot of color-coded functional connectivity of largest 100 values selected during the 7 h of basal recording (i.e., 7 h) for two representative experiments. Top panel: Control experiment did not show variation with respect to the spontaneous condition. In contrast, CCh application caused a complete loss of correlation in the first 1 h with respect to the basal condition, and it did not recover after a few hours (right panels). Bottom panel: Bar plot of largest 100 STTC for non-treated cultures (*N* = 4) in the basal (gray bar) and in the control (blue bar) condition. Each bar corresponds to one of three different time points of an hour: start, middle, and end for each experimental condition. In the middle, plot of largest 100 STTC for treated cultures (*N* = 7) in the basal (gray bar) and during the CCh administration (red bar). On the right, box plot of largest 100 STTC for the treated experiment evaluated in four different time-point. During the 24 h of CCh application, the SSTC values decreased significantly respect to the 7 h of the basal recording (gray box). White arrow indicates the starting point for CCh treatment. **(C)** On the left, bar plot of the percentage of STTCs values that significantly decreased (above 20%) respect to the basal in the control condition (blue bar, *N* = 4) during three different time points (start, middle and end). In the middle, bar plot of the percentage of STTCs values that significantly decreased (above 20%) respect to the basal phase during the CCh administration (red bar, *N* = 7). On the right, bar plot of the percentage of deleted connections (i.e., STTC) during the CCh application. For box plot **(B,C)**, the small square indicates the mean, the central line illustrates the median and box limits indicate the 25th and 75th percentiles. Whiskers represent the 5th and the 95th percentiles. Statistical Analysis was carried out using Mann–Whitney comparison test, ^**^p < 0.01.

Following the previous results, we analyzed the changes of correlation upon CCh administration (see Section Materials and Methods). The effects of CCh on network activity correlation were visible when comparing the functional connectivity maps at different time points (Figure 3A). CCh caused an initial loss of correlation, resulting in 80% decreased of mean STTC values with respect to the basal condition, followed by a partial recovery after a few hours (Figure 3B). During the control experiment, only 2% of the STTC values significantly decreased at the end of the recording but the number of links did not change with respect to the basal condition (Figure 3C). In the first few hours following CCh application, the 98% of STTC value significantly decrease with respect to the basal value. Moreover, the 5% of the STTC was deleted after the treatment and not recovered after 24 h.

**Figure 4:**
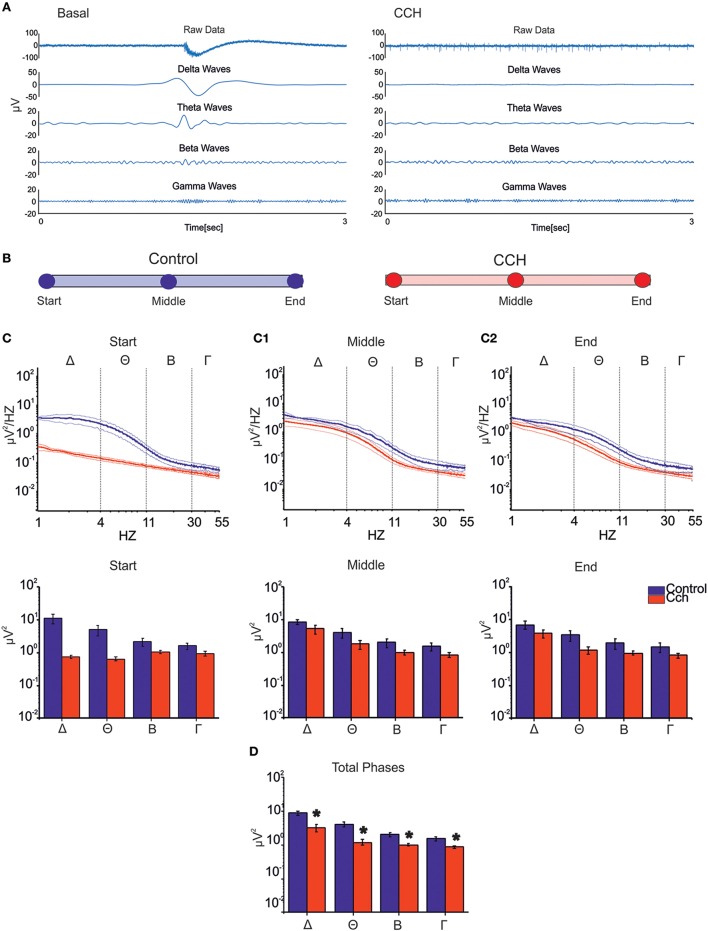
**LFP analysis. (A)** Signal filtered to specific frequency bands involved in the sleep for one channel of a representative experiment. The CCh treatment induced a suppression of the low waves delta (1–4 Hz), theta (4–11 Hz), beta (11–30 Hz), and gamma (30–55 Hz) respect to the control condition. **(B)** Different time points (1 h long) considered for each two types of experimental condition. **(C)** PSD during the control phase (*N* = 2, blue line) and the CCh application (*N* = 4, red line) in the first 1 h of recording (Top Panel). Bold lines and shaded regions correspond to mean ± SEM. Column bar of integrated PSD per frequency bands in the same time point (Bottom Panel). CCh treatment caused a suppression of the low components of the signal (1–55 Hz). **(C1)** PSD after 12 h of recording (Top Panel). Column bar of integrated PSD per frequency bands in the same time point (Bottom Panel). **(C2)** PSD in the last hour of recording (Top Panel). Column bar of integrated PSD per frequency bands in the same time point (Bottom Panel). **(D)** Column bar of integrated PSD evaluated through the key time points described in **(B)**. The decrease involves in particular the delta and theta frequency bands. **(D)** Bar plot of the power for all phases in the different frequency ranges for the control experiment (*N* = 2; Delta Power = 8.87 ± 1.36 μV^2^; Theta power = 4.15 ± 0.71 μV^2^; Beta power = 2.03 ± 0.29 μV^2^; Gamma power = 1.53 ± 0.19 μV^2^) and for the experiment with the administration of CCh (*N* = 4; Delta Power = 3.28 ± 0.82 μV^2^; Theta power = 1.21 ± 0.23 μV^2^; Beta power = 1.00 ± 0.07 μV^2^; Gamma power = 0.87 ± 0.07 μV^2^). Statistical analysis was carried out using Mann-Whitney test, ^*^p < 0.01.

All main waves (i.e., delta, theta and partly beta), which characterize classical sleep-wake-cycle, are strongly suppressed upon CCh treatment (Figure 4A, right panel). Thus we evaluated how the power spectral density (PSD) changed at different time points during both control and CCh protocol (Figure 4B). The control experiments indicate that most power is concentrated in the delta frequency band, reflecting very slow oscillations in the LFP that match the appearance of synchronous bursting activity in the MUA (Figure 4C, blue line). PSD trend remained stable throughout the recording in the control experiments, whereas CCh application to the culture caused a marked decrease of the low-frequency power in the LFP (especially in the delta and theta range; Figure 4C, red line). The total power in the first hour (i.e., Start; Figure 4C) decreased by 93% in delta, 87% in theta, 50% in beta, and 40% in slow gamma bands with respect to the control experiment. After 12 h from the treatment (i.e., Middle; Figure 4C1), the integrated power in the delta and theta bands shows a partial recovery especially with respect to the first hour: the total reduction drops to 37% for delta and 55% for theta bands. The average PSDs (when considering all phases) globally show marked reduction of power in all frequency bands caused by CCh (62% for delta; 71% for theta; 50% for beta; 43% for gamma) (Figure 4D).

**Figure 5:**
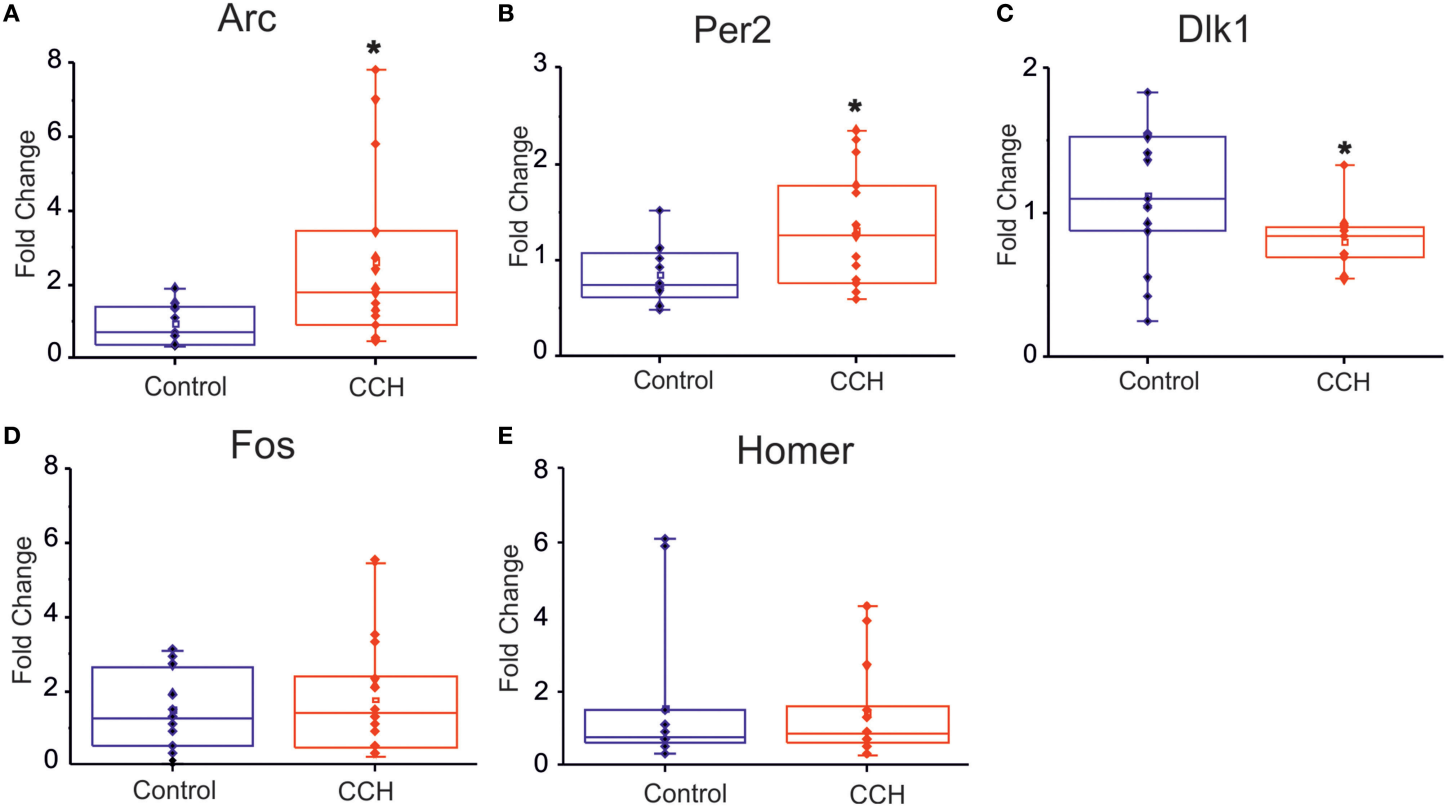
**Gene expression analysis following CCh treatment. (A–C)** Box plot of Arc, Per2, Dlk1, Fos, Homer1 mRNA levels in primary neuronal rat cultures, treated at DIV 30 with 20 μM of CCh or with normal medium as control. For each box plot **(A–E)**, the small square indicates the mean, the central line illustrates the median and box limits indicate the 25th and 75th percentiles. Whiskers represent the 5th and the 95th percentiles. Raw data are plotted as blue and red dots, respectively for control and CCH treatments. The dots represent data from three different experiments. ^*^p < 0.05 after Student.

We complemented the electrophysiological study with a gene expression assessment. In particular, we investigated whether the network physiological effects of CCh treatment were corroborated by a gene expression modulation that recapitulated the clock-like and the homeostatic-like aspects of sleep. We performed gene expression analysis of DIV 30 primary rat neurons treated with 20 μM of CCh for 24 h and harvested 24 h later. Interestingly, two classical immediately early genes (IEG), *Arc* and *Fos* (Figure 5D), were upregulated by CCh, *Arc* being significantly overexpressed (*p* = 0.02 control *n* = 13, CCh treated *n* = 14; Figure 5A), while *Homer1a* was not altered (Figure 5E). Moreover, we observed a 50% significant (*p* = 0.02 control *n* = 12, CCh treated *n* = 15) increase of *Per2* gene expression in cultures treated with CCh compared to control cultures (Figure 5B). In addition to the classical markers of the circadian and the homeostatic control of sleep, we tested new genes that play a role in the epigenetic regulatory processes of sleep. In particular, we found that *Dlk1* (Figure 5C), a genomic imprinted gene, was significantly (*p* = 0.03 control *n* = 15 CCh treated *n* = 13) downregulated (i.e., by ~30%) by CCh treatment compared to controls.

## Discussion

In this study we presented electrophysiological and genetic evidence that primary cortical cultures, usually displaying synchronized low-frequency firing patterns under spontaneous conditions, are able to encompass some essential features of sleep in a controllable way. LFP analysis complemented MUA analysis and allowed us to look for classical sleep electrophysiological signatures (i.e., slow waves) in our simplified *in vitro* experimental model. In fact, we found that under spontaneous condition most LFP power was actually confined to the delta frequency range, as it can be usually observed in the EEG signal during NREM phase in sleeping rodents (Steriade et al., 1993). In addition, *in vivo* these waves were found to be negatively correlated with the response to arousing stimuli (Neckelmann and Ursin, 1993). These observations suggest that cortical cultures are usually characterized by a sleep-like state, confirming recent data from several different laboratories (Sengupta et al., 2011; Hinard et al., 2012). Since cortical cultures spontaneously lack sleep-wake rhythm that occurs in *in vivo* systems, we tested whether we could mimic wake-like states by chemical manipulation, in order to use this as a simplified and easily controllable experimental model to investigate sleep more completely. The application of CCh resulted to be an efficient experimental strategy to suppress classical sleep-like properties of activity, resulting in an asynchronous firing pattern with many isolated spikes. In fact, while the percentage of isolated spikes increased significantly upon CCh stimulation, the burstiness level markedly decreased. Similarly, the correlation of activity dropped in the first 1 h after CCh treatment, while the firing rate increased. The value of STTC remained significantly low during the entire recording. The percentages of decreased STTC values remain higher during the 24 h of the CCh application. Moreover, the administration of CCh induced a suppression of delta and theta waves, a range encompassing the entire spectrum of sleep *in vivo*. These changes testify for a significant alteration of sleep-like states in the network but, surprisingly, beta and low-gamma waves, which are typically present during wakefulness and REM sleep, do not show power variations compared to the low frequency components of the signal. This study demonstrates that wake-like and sleep-like electrophysiological signatures can be dissociated *in vitro* and, possibly studied independently within this experimental model. Furthermore, we demonstrated that CCh alters specific sleep-like signatures.

As a complement of the above conclusions, gene expression profile in our study confirmed an opposite effect on the circadian and the homeostatic components of sleep-like states. Our results demonstrated that CCh exerts a significant role in activating molecular markers of sleep-wake rhythms, for example *Per2* expression. Yet, we showed that classical sleep-dependent IEGs (Cirelli et al., 2006) are also essential in neuronal responses of our *in vitro* experimental model. However, *Homer1a*, a gene widely considered as the main molecular marker of the homeostatic control of sleep (Hinard et al., 2012), was not affected by the treatment. This raises the issue that this *in vitro* model is able to reproduce only partially the range of homeostatic phenomena that characterize the regulation of sleep. Moreover, it raises also a new possibility: can we use *in vitro* experimental model to dissect molecular markers of homeostatic and circadian control of sleep? Indeed, *PER2* is a marker of the circadian control of sleep (Kopp et al., 2002; Shiromani et al., 2004) while Homer1a is an important marker of the homeostatic process of sleep. In addition we have shown that *Dlk1* is a molecular marker of sleep-like electrophysiological aspects *in vitro*, confirming recent evidence *in vivo* (Tinarelli et al., 2014).

Altogether our results demonstrate that our experimental model in cultures is not able to reproduce an awake-like state, however it is a promising model to manipulate and study sleep properties in a very controlled way.

In conclusion, this study shows that MEA recordings coupled to cortical cultures represent a favorable model to investigate the essential neuronal network features of sleep *in vitro*. We envisage that sleep-related electrophysiological phenotypes can be assessed by using *in vitro* experimental models. In particular, the combination of new gene editing technologies such as CRISPR/cas9 system and MEA recordings has the potential to accelerate the investigation of loss or gain of function due to gene mutations in sleep studies.

## Author contributions

IC, MC, and VT designed the work. IC performed the electrophysiology experiments. FT performed the gene expressions. IC, FT, VP, and MC analyzed the data. IC, FT, VT, and MC wrote the manuscript. IC, VP, VT, and MC revised the manuscript. MC and VT supervised the study and equally contributed.

### Conflict of interest statement

The authors declare that the research was conducted in the absence of any commercial or financial relationships that could be construed as a potential conflict of interest.
