# Corrigendum: A Simplified *In vitro* Experimental Model Encompasses the Essential Features of Sleep

**DOI:** 10.3389/fnins.2016.00409

**Published:** 2016-09-07

**Authors:** Ilaria Colombi, Federico Tinarelli, Valentina Pasquale, Valter Tucci, Michela Chiappalone

**Affiliations:** ^1^Department of Neuroscience and Brain Technologies, Istituto Italiano di TecnologiaGenova, Italy; ^2^Dipartimento di Neuroscienze, Riabilitazione, Oftalmologia, Genetica e Scienze Materno-Infantili (DINOGMI), Università degli Studi di GenovaGenova, Italy

**Keywords:** cortical culture, microelectrode arrays, homeostasis, spike train analysis, local field potentials, gene expression

Reason for Corrigendum:

The primer pairs for the gene Homer1 reported in the original manuscript referred to the longer isoforms Homer1b/c and not Homer1a. Moreover, we added some missing references. Therefore

at Page 2, Third paragraph, 11th line: the reference (Tateno et al., 2005; Corner, 2013) is corrected with (Tateno et al., 2005; Kaufman et al., 2012; Corner, 2013). The authors apologize for the missing reference. This error does not change the main scientific conclusions of the article;

at Page 5, Table 1: *Homer1a is corrected with Homer1b/c*. The authors apologize for the mistake. This error does not change the main scientific conclusions of the article;

at Page 9, Third paragraph, 11th line: *Homer1a is corrected with Homer1b/c*. The authors apologize for the mistake. This error does not change the main scientific conclusions of the article;

at Page 9, Third paragraph, 16th line: the phrase “In addition to the classical markers of the circadian and the homeostatic control of sleep” is corrected with “In addition to the classical markers of the circadian and the synaptic homeostasis control of sleep.” The authors apologize for the mistake. This error does not change the main scientific conclusions of the article;

at Page 9, Second paragraph, 1st line: the phrase “As a complement of the above conclusions, gene expression profile in our study confirmed an opposite effect on the circadian and the homeostatic components of sleep-like states” is corrected with “As a complement of the above conclusions, gene expression profile in our study confirmed an opposite effect on the circadian and the synaptic homeostatic components.” The authors apologize for the mistake. This error does not change the main scientific conclusions of the article;

at Page 9, Second paragraph, 10th line: the phrase “However, Homer1a, a gene widely considered as the main molecular marker of the homeostatic control of sleep (Hinard et al., 2012), was not affected by the treatment” is corrected with “However, Homer1b/c, a gene considered as a molecular marker of the synaptic excitability response to stimulation (Ango et al., 2000; Nakano-Kobayashi et al., 2014; Cao et al., 2015), was not affected by the treatment.” The authors apologize for the mistake. This error does not change the main scientific conclusions of the article;

at Page 9, Second paragraph, 17th line: the phrase “can we use *in vitro* experimental model to dissect molecular markers of homeostatic and circadian control of sleep? Indeed, PER2 is a marker of the circadian control of sleep (Kopp et al., 2002; Shiromani et al., 2004) while Homer1a is an important marker of the homeostatic process of sleep” is corrected with the phrase “can we use *in vitro* experimental model to dissect molecular markers of synaptic homeostasis and circadian control of sleep? Indeed, PER2 is a marker of the circadian control of sleep (Kopp et al., 2002; Shiromani et al., 2004) while Homer1b/c is an important marker of neuronal excitatory synapses activity.” The authors apologize for the mistake. This error does not change the main scientific conclusions of the article.

## Author contributions

Original Research article: IC, MC and VT designed the work. IC performed the electrophysiology experiments. FT performed the gene expressions. IC, FT, VP, and MC analyzed the data. IC, FT, VT, and MC wrote the manuscript. IC, VP, VT, and MC revised the manuscript. MC and VT supervised the study and equally contributed. Corrigendum: FT and VT wrote the corrigendum. MC, IC, and VP revised the corrigendum. All authors approved the final version.

### Conflict of interest statement

The authors declare that the research was conducted in the absence of any commercial or financial relationships that could be construed as a potential conflict of interest.
